# My inner experience

**DOI:** 10.4103/0019-5545.31590

**Published:** 2006

**Authors:** B.K. Garg

I am a postgraduate from the science and management streams from premier educational institutes of India. I had to struggle hard for this education and I got to work with the best of companies/employers in India. I have lived for extended periods (6–9 months) in Germany and Australia and have also been to Ethiopia (Africa) for 15 days. My childhood was peaceful with father working in the government service. However, my mother was a chronic patient of schizophrenia for 20 years. She has recently expired.

Essentially, I have had good education and life. However, I experienced 3–4 bouts of schizophrenia during the past 6–7 years. I am clear in my heart and mind that these were results of some sort of malfunctioning of the mind or more precisely of the thought process. As far as the concept of split-thinking is concerned, yes I had split-thinking during all these instances. Thus, while speaking one sentence I used to throw up three meanings. Today I can clearly look back and recall all my foolish thinking during those periods of illness. Thinking and activities done during the periods of illness keep coming back to me. Essentially, what the medical science calls, I had enjoyed my illness. Yes, I have had one sure trait of this illness that I was—and perhaps still am—very ambitious. During the illness, I used to think that I was the central person in the whole universe and that things such as relatives, neighbours, TV, radio, the sun, moon and stars were all operating under a clear design to achieve some divine purpose. There used to rotate some kind of a circle of charge in my mind, in alternating directions—clockwise and anticlockwise—depending upon the positive or negative direction of thinking. I used to interpret the talks and sayings of people including written things in a strange way—a mix of English and vernacular languages.

But what baffles me is that during each incidence of illness I have had some supernatural experience and I became hyperactive, both physically and mentally. The first time, when the illness began, I was sleeping peacefully and I saw a Goddess (*Devi*) surrounded by flames and made up of electric charge moving before my face, which entered my forehead. The figure of the Goddess made up of electric charge was quite realistic unlike the scenes during my dreams. I got up and found myself shivering. I switched on the electric heater and felt as if my whole body was absorbing charge through my feet and palms. I went to the balcony and saw a very bright star on the top of the moon (three-fourths in size). I then went into the mode of schizophrenia thinking. One day, I was resting against the wall looking at an incandescent bulb and suddenly the charge got absorbed in my face and was circulating in my jaws and forehead. I felt immense strength in my body. Also, during this period I used to see a cosmic rain or showers of rays coming from the ceiling made of concrete. My hearing had become acutely loud and I would hear the cosmic noise as that of fast blowing wind. I would hear the neighbours talking and interpret it as if the whole universe was conversing with me. The peacocks in the nearby forest and the neighbours all seemed to influence me. As if by intuition, I opened the electric switch plates and I found a newspaper dipped in whitewash having strange stories in the vernacular language, which I would try to read and interpret. Similarly, with the wall clock, where I found balls of newspaper. There was a red LED (light emitter) in one of the switches in the wall and I suddenly learned that with my concentration I could flicker that LED. I started concentrating on the tubelight and the ultraviolet bulb in the tubelight's starter would emit charge, which I used to absorb in my forehead and the tube's light would automatically switch on and off with the concentration of mind. I used to clearly see the charged particles in the ray of light. I had a small incandescent bulb in a small electric holder and for hours I would concentrate on the filament with closed eyes. On closing eyes I would see different changing patches of light yellow, green turning to red and finally a black light flame, which would hover before my eyes and then get absorbed in my eyes. One day while concentrating I saw a red dot/circle which performed simple harmonic motion along a straight line. During the illness I would try to somewhat naturally overcome the basic need of hunger and I would eat very little. I became very thin and energetic like sadhus and I started growing beard and wearing my hair long. One day two rays of light honed my both eyes like a honing tool having clear axis at two points each in each ray.

On another occasion I saw in the sunlight a very small black spider moving/jumping on my fingers like the spiderman, which after some time melted away in the sunlight. On yet another occasion, I saw thick charge-like small stars floating in the air, which would come and get absorbed in my eyes. Once I had a ray of ultraviolet light emitting from my forehead onto a spider on the ceiling with my mind asking questions and getting yes/no in the form of trance of the spider.

I have been told by the doctor that excess of dopamine causes this accumulation of charge between the nerve endings in the brain. But somewhere in my inner mind I feel there is some religious angle to it. During my stay in Germany I saw documentaries about the head or the brain and the halo surrounding it. Energy dissipation occurs through the hair of the body and that perhaps explains why most sadhus wear their hair long.

I have given an honest account of what transpired with me during my bouts of illness. Perhaps this can throw some light on this wicked illness, which pulls out a person from the social mainstream and makes him or her a social recluse or self-confined.

I have been treated for my illness 2–3 times and each time I have recovered fully. Since my last relapse I have been under treatment from the Institute of Human Behaviour and Allied Sciences, New Delhi. On the persuasion and advice of my treating doctor, I have gathered courage to share my experiences with the readers. The wonder drug is risperidone. Presently, I am on continuous medication for the past 1 year and keep wondering when I will be off medicines. Also, I fear that on stopping the medicines I will have a relapse. I guess doctors know best and I depend on their advice and treatment.

